# Regenerative Engineering: Current Applications and Future Perspectives

**DOI:** 10.3389/fsurg.2021.731031

**Published:** 2021-11-03

**Authors:** Dana Goldenberg, Caroline McLaughlin, Srinivas V. Koduru, Dino J. Ravnic

**Affiliations:** ^1^Irvin S. Zubar Plastic Surgery Research Laboratory, Penn State College of Medicine, Hershey, PA, United States; ^2^Department of Surgery, Penn State Health Milton S. Hershey Medical Center, Hershey, PA, United States

**Keywords:** regeneration, engineering, surgery, tissue dysfunction, transplantation

## Abstract

Many pathologies, congenital defects, and traumatic injuries are untreatable by conventional pharmacologic or surgical interventions. Regenerative engineering represents an ever-growing interdisciplinary field aimed at creating biological replacements for injured tissues and dysfunctional organs. The need for bioengineered replacement parts is ubiquitous among all surgical disciplines. However, to date, clinical translation has been limited to thin, small, and/or acellular structures. Development of thicker tissues continues to be limited by vascularization and other impediments. Nevertheless, currently available materials, methods, and technologies serve as robust platforms for more complex tissue fabrication in the future. This review article highlights the current methodologies, clinical achievements, tenacious barriers, and future perspectives of regenerative engineering.

## Key Points

- Regenerative engineering is an emerging field that combines tissue engineering with material science, stem cell biology, and developmental biology to create complex tissue constructs.
- A variety of stem cell sources are often used as the starting platform.
- An engineered three-dimensional microenvironment supports cellular differentiation into functional tissue.
- An assortment of manufacturing processes can facilitate tissue assembly.
- Bioengineered constructs often provide a superior model for preclinical disease and drug response modeling.
- While early, regenerative engineering is underway in the clinical domain, though its successes are predominantly limited to thin or avascular structures.
- Advances are rapidly being realized and subsequent clinical translatability will broaden.
- Surgeons should become acquainted with these emerging technologies.

## Introduction

Tissue loss and organ dysfunction are commonplace in modern medical care. Over the past half century, the free transfer of tissue and organ transplantation has revolutionized our ability to care for these diverse entities. This spans the spectrum from breast reconstruction following mastectomy to solid organ transplants and most recently composite tissue allotransplantation. Despite the advances, these complex surgical strategies are not without many problems. All these approaches require a suitable donor source, which is often lacking at both the autologous and allogenic level. Autologous tissue transfers often have significant donor site morbidity and organ transplants require lifelong immunosuppression. Both approaches can suffer devastating effects from a thrombotic event. Furthermore, there still exist pathologies which are untreatable (1). Therefore, the need for engineered tissue and organ replacements permeates virtually every surgical specialty from reconstructive to transplant surgery (2).

Tissue engineering represents an ever-growing interdisciplinary field aimed at creating biological replacements which can mimic native tissue (3–5). Regenerative engineering has been described as the amalgamation of tissue engineering with material science, stem cell biology, and developmental biology (6). By combining cells, materials, and bioreactive molecules along with a proper manufacturing platform; regenerative engineering is slowly being realized. Ideally, these replacement tissues could be built on an autologous platform which would offer personalized treatment options. However, there are numerous hurdles to overcome until ubiquitous clinical translation. Nevertheless, there have been some successes in the field, both in the research and clinical arenas which provide a stable foundation for further advances (7–9). Here we review the basic aspects of the regenerative engineering platform along with some preclinical and clinical successes.

## Stem Cells

**Figure 1:**
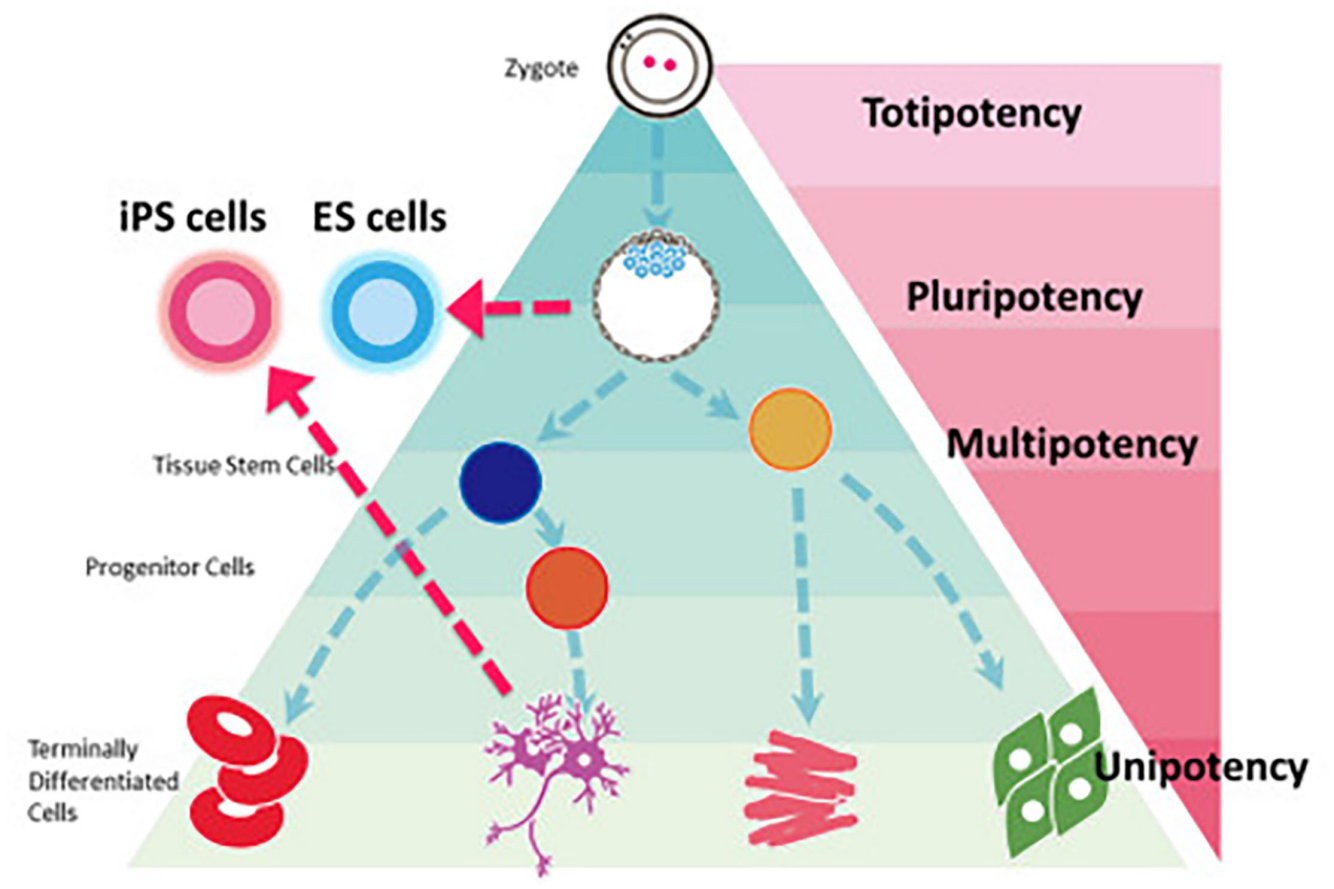
Stem cell classification based on potency. iPS cells can be reprogrammed from a fully differentiated cell to regain the pluripotency seen in embryonic stem cells. Most regenerative engineering applications focus on the use of either iPSCs or multipotent stem cells. Reprinted Sugawara et al. (11).

There have been tremendous advancements in cell sourcing for regenerative engineering over the past two decades, varying in their derivation and differentiation ability. This includes both adult cells and stem cells. Since, stem cells are capable of both self-renewal and differentiation, they have found a distinctive niche role in tissue engineering applications (10). Stem cells can broadly be defined as being totipotent, pluripotent, and multi-/oligo-/unipotent (Figure 1, Table 1) (11).

**Table 1:** Summary of stem cells.

**Stem cells**	**Description**	**References**
Totipotent stem cells	• Gives rise to all three germ layers as well as extraembryonic tissues • Available only during early embryogenesis • Not accessible for engineering applications	(9, 10)
Pluripotent stem cells	• Gives rise to all three germ layers • Can be induced from fully differentiated cells via reprogramming • Gold standard for stem cell-based therapies • Widely investigated across a multitude of tissue types	(11–40)
Multi-, Oligo-, or Unipotent stem cells	• Present postnatally • Identified in many tissues • Limited in differentiation potential • Can be easily accessible (i.e., adipose) • Widely investigated in regenerative engineering applications	(41–69)

### Totipotent Stem Cells

Totipotent stem cells can give rise to all three primary germ cell layers, as well as, extraembryonic tissues, such as the placenta. This means that they are only present in the first weeks of embryogenesis (12, 13). As such, they are inaccessible and unavailable for tissue engineering applications.

### Pluripotent Stem Cells

Pluripotent stem cells are present in later embryogenesis, therefore while they similarly can differentiate into all three germ layers, they are unable to differentiate into the placenta (14). Widely recognized as embryonic stem cells (ESC), they represent the gold-standard in stem cell-based therapies because of limitless differentiation capability and self-renewal. Since being described over two decades ago (15), ESCs have significantly contributed toward the understanding of stem cell biology, developmental processes, and the translatability of regenerative endeavors. They have been used in various clinical trials such as macular degeneration, cardiac repair, and diabetes (16, 17). Additional experimental studies are investigating therapeutic utility for lung disease (18), and ischemic pathologies (19). Furthermore, with human ESCs, the ability to study disease specific mutations has expanded greatly. There have been over 100 human embryonic stem cell lines registered for this purpose (20), investigating wide-ranging congenital ailments. Despite tremendous scientific advancements, ESCs continue to be plagued by significant disadvantages. An autologous option is not available with ESCs and there exists the unwanted effects of possible immune rejection after allogenic grafting (21). Human ESCs have traditionally been grown utilizing a murine fibroblast feeder layer with potential exposure to mouse retroviruses (22), although this has been remedied. *In vitro*, ESCs spontaneously differentiate into embryonic bodies but can form teratomas during animal implantation. In addition, karyotype alterations can appear following prolonged culture with highly-passaged cells forming less mature tumors (23). The potential for tumor formation is a significant concern toward clinical translatability. Furthermore, long-term clinical sustainability of these efforts is uncertain secondary to ethical concerns, which continues to be the largest disadvantage of this type of therapy, as human embryo destruction is requisite to the generation of ESCs. These concerns have not diminished with time and there continues to be discordance between the protection of embryonic life vs. the potential to remedy postnatal ailments and alleviate suffering (16). Accordingly, this type of work is subject to significant regulations and even prohibited in some countries, while being threatened by frequent changes in governmental policies (24). In multicultural environments, such as the United States, the respect of various religious entities will need to be balanced to allow for the progress of human ESC research. This will only serve to complicate the discussion between physicians and patients regarding ESC-based treatments, while attempting to formulate a consensus on “how much manipulation can be allowed until it is considered playing God and morally unacceptable?” (25). Nonetheless, because of their differentiation ability, these pluripotent stem cells are extremely valuable to tissue engineering and regenerative medicine.

Fortunately, Shinya's Yamanaka's reprogramming strategy has permitted an alternative route for the widespread use of pluripotent stem cells (26, 27). Yamanaka has obviated the ethical concerns of ESCs by reprogramming fully differentiated fibroblasts into induced pluripotent stem cells (iPSCs) using transcription factors Oct3/4, Sox2, c-Myc, and Klf4 under embryonic stem cell culture conditions (28). It has also now been demonstrated that multiple differentiated adult cell varieties have the potential for reprogramming into a pluripotent state (29, 30). iPSCs seem to offer the benefit of pluripotency without the wide-ranging ethical quandaries associated with ESCs. Comparisons with the gold standard ESC has demonstrated significant similarities with regard to transcriptional profiles and differentiation potentials (31). This has generated much excitement since being first described in 2006 (26). iPSCs have been extensively researched and utilized in regenerative medicine and tissue engineering (10, 32–38). Additionally, iPSC clinical translation has recently been demonstrated in replacing eye tissue damaged by age related macular degeneration, suggesting safety (39). The advantages of programmed pluripotency are only heightened by the potential to use autologous cellular material which can mitigate against immune rejection.

Despite these recent advances there are still numerous disadvantages associated with iPSCs that need to be considered. For example, nearly $900,000 USD was needed to develop and test the iPSCs for the first clinical trial, mentioned above (40). Furthermore, no consistent protocols have been established that yields the induction of iPSCs with high efficiency. Likewise, the safety profile of iPSCs is still a concern, especially with numerous cellular starting sources and programming schemes. The development of new Next Generation Sequencing technologies has further demonstrated the potential for genomic instability in iPSCs (41) raising fear of tumor progression following implantation. Although ethical concerns seem less intense than those seen with ESCs, they are still somewhat prevalent. For example, some claim that iPSCs could, in theory, be used to create human embryos, and therefore may be as problematic as human ESCs (42). It would then need to be established if these embryos would be subject to the same ethical and legal implications regarding humanity and protection. Also, there are apprehensions with the ability to clone human beings and to produce germ cells (43). However, it is likely that guidelines can be formulated to address these concerns allowing for improved clinical translation of regenerative engineering innovations.

### Multi-, Oligo-, or Unipotent Stem Cells

Adult stem cells (ASC) are typically described as unipotent, oligopotent, or multipotent cells that persist into the postnatal period (44). These stem cells have the capacity for self-renewal and to develop into individual or multiple cell types within a specific tissue or organ. Their persistence into adulthood offers them significant potential for clinical therapeutics. In fact, over the past four decades, patients afflicted by blood disorders have been treated with adult stem cells via bone marrow transplants (45, 46). While clinical use of these hematopoietic stem cells has become widespread, the true potential of adult stem cells has yet to be realized, but they also have found an enlarged role in regenerative engineering endeavors (47–58).

Adults stem cells have been identified in many tissue types including those of mesenchymal (59), intestinal (60), neural (61), and endothelial (62) origin. While they lack the diverse pluripotency seen with embryonic or induced pluripotent stem cells, their differentiation potential is still impressive and is continually being explored. For example, adipose derived stem cells (ADSCs) represent a type of mesenchymal stem cell that can easily be accessed and retrieved through a simple minimally invasive procedure (63). While their potential was initially identified for mesodermal replacement therapy (64), there has been some suggestion that they may have the ability to transdifferentiate into endodermal (65) and ectodermal (66) lineages, further expanding their therapeutic potential. With adipose excision (ADSCs) or other interventions, such as childbirth (umbilical cord stem cells); adult stem cell rich tissue could be utilized for the generation of biobanks (67). This represents a significant advantage, in that tissue that is typically discarded can yield large numbers of stem cells for either autologous or allogenic use. Thus, replicating what is currently done for packed red blood cells, platelets, and fresh frozen plasma. Specifically, it is believed that mesenchymal stem cells offer low immunogenicity (68) and are immunomodulators (69); thus allowing for allogenic transplant and pooled donors. Minimal immunogenicity has been observed across multiple mesenchymal stem cell types including umbilical cord, placenta, and dental pulp (70). While clinical translation is the desired endpoint, adult stem cells are ideal for human *in vitro* testing platforms such as disease modeling, drug screening, therapeutic efficacy, and organoid regeneration.

Disadvantages of adult stem cells are focused on the decrease in pluripotency along with the lack of continued self-renewal. Furthermore, the retrieval of ASCs can be plagued by problems with cellular cross-contamination. For example, the dissociation of adipose tissue to obtain the stromal vascular fraction and subsequent cell sorting does not guarantee a pure ADSC aliquot. In addition, the need for cellular expansion in long-term cultures may lead to bacterial contamination. However, the most profound benefit of adult stem cell sources is the ability to offer therapy that is not overshadowed by ethical disputes. While adult stem cells do not face the immense ethical dilemma of embryo destruction with associated legal and religious guidelines, there are still some noteworthy scenarios where principled practice must be developed. As ASCs are highly varied, widespread, and easily retrieved the potential for biobanking; associated abuse, has come to the forefront. Accordingly, issues of consent, control, and justice will need to be examined (71). In addition, with the potential for long-term self-renewal there is the troublesome concern of loss of patient privacy and identity. As biobanks are for-profit business endeavors stem cell donor compensation models may need to be established. However, these ethical dilemmas are more likely to achieve a consensus agreement than those which surround ESCs and iPSCs, leading many to believe that ASCs represent the most streamlined path toward large scale clinical translation. Engineered translation may become even more appealing as the true pluripotency of stem cells in adult tissues continues to be investigated and expanded (72).

While many researchers have a preference, there is currently no consensus for the best cell source for regenerative engineering, as each pose unique advantages and disadvantages. However, it is undeniable that our pool of cellular options has dramatically improved over the past two decades and represents a solid footing for further advances in tissue engineering and regenerative medicine.

## Materials and Bioreactive Molecules

Although, cell sourcing is integral to tissue engineering, its organization and microenvironment determines ultimate success (73). Therefore, supporting materials and molecules are needed to create the proper microenvironment for cell proliferation and differentiation into functional tissue. This is often achieved by suitable scaffolding and enabling trophic biomolecules. These important factors provide durability to tissue-engineered constructs by facilitating growth, induction, and long-term maturation (5). Suitable scaffolds aim to mimic native tissue, providing a framework for cell attachment, migration, growth, and differentiation, while allowing cells for reorganization into a functional 3D network (5, 74).

Until recently, most cell cultures had been performed in two-dimensions on flat and rigid materials. While useful, they fail to replicate the complex three-dimensional architecture found in living organisms. Therefore, the information obtained from them often does not translate to clinical success (75). This becomes most significant in regenerative medicine applications where the substitution part needs to function in three dimensions, such as a vascular graft or bone replacement. Therefore, it is not surprising that 3D cultures and scaffolds would be desired for these applications. 3D culture systems encompass cells grown on a matrix, within a matrix, or grown in suspension. With all these approaches, cells maintained in 3D culture differ both morphologically and physiologically compared to 2D (Table 2) (75).

**Table 2:** Key differences between 2D and 3D culture systems.

**2D culture**	**3D culture**
Monolayer cell growth on plastic or glass	Natural cell growth on soft materials like collagen or other biomaterials
Easy fabrication	Additional expertise needed to make scaffolds and suspend cells
All cells equally exposed to nutrients and oxygen	Innermost cells may be deprived of nutrients and oxygen leading to necrosis
Gene expression profiles are dissimilar to *in vivo* tissues	Gene expression profiles are more like *in vivo* tissues
Are not predictive of the *in vivo* effectiveness/toxicity of drug treatments	Better predictors of *in vivo* drug effectiveness/toxicity

Scaffolds/matrices used for 3D culture can be fabricated from a variety of techniques, being either biologically or synthetic based. When determining the suitability for regenerative engineering applications; biocompatibility, biodegradability, mechanical properties, manufacturing technology, and scaffold architecture need to be considered. Ideally, the scaffold can provide a framework and initial support for cell attachment, proliferation, and differentiation, ultimately forming an extracellular matrix (ECM) (76). The 3D environment facilitates complex cell-to-cell and cell-ECM interactions, mimicking what occurs *in vivo*; and cells tend to secrete more ECM in this setting. Accordingly, a wide range of cellular processes are impacted including proliferation, differentiation, morphology, gene expression, protein synthesis, and response to stimuli (75). As cells in 3D conditions aggregate, a diffusion gradient occurs where the innermost cells may proliferate under slightly different circumstances than those on the periphery (77). This will affect oxygen delivery and metabolite removal, both *in vitro* and *in vivo*. However, these conditions can be refined by controlling the porosity of the scaffold, which will also impact cell penetration and ingrowth, and may augment vascularization upon implantation (78). Scaffold porosity can also be used to impact the overall stiffness which may be beneficial as mechanical functionality will require a dense scaffold while other processes will benefit from a more porous one (79). Furthermore, scaffold stiffness has been found to have a substantial effect on stem cell differentiation potential (80).

In most native tissues, the ECM serves as this scaffold, providing structural integrity, functionality, and ideal conditions for facilitating cell growth (81, 82). Inspired by this natural, multi-tissue construct, various materials have been proposed for creating these engineered scaffold designs (2). Many of these designs utilize hydrogels which form a biochemically and mechanically appropriate environment for cell deposition and growth (2). The choice of material used is highly dependent on the mechanical and structural requirements of the construct, which differs based on the desired tissue product (83). However, to be functional for tissue engineering, they must be mechanically stable, non-toxic, and have an appropriate degradation rate (2). Organic polymers can be isolated from animal or human tissue and offer the advantage of inherent bioactivity and similarity to native ECM (83, 84). However, while they are more cell friendly, their mechanical properties are weak (85). Synthetic hydrogels have stronger mechanical and structural properties because of their ability to be tailored by chemical modification which may better suit them for these applications (2, 83). However, they have poorer biocompatibility, with increased chances of toxic degradation and loss of mechanical properties during tissue degradation *in vivo* (83). Both types of scaffolds are also able to be constructed either *in vivo* by implantation or *in vitro* in a bioreactor followed by implantation (5). Each scheme has benefits and detriments, and the better option depends on the specific tissue being constructed.

**Figure 2:**
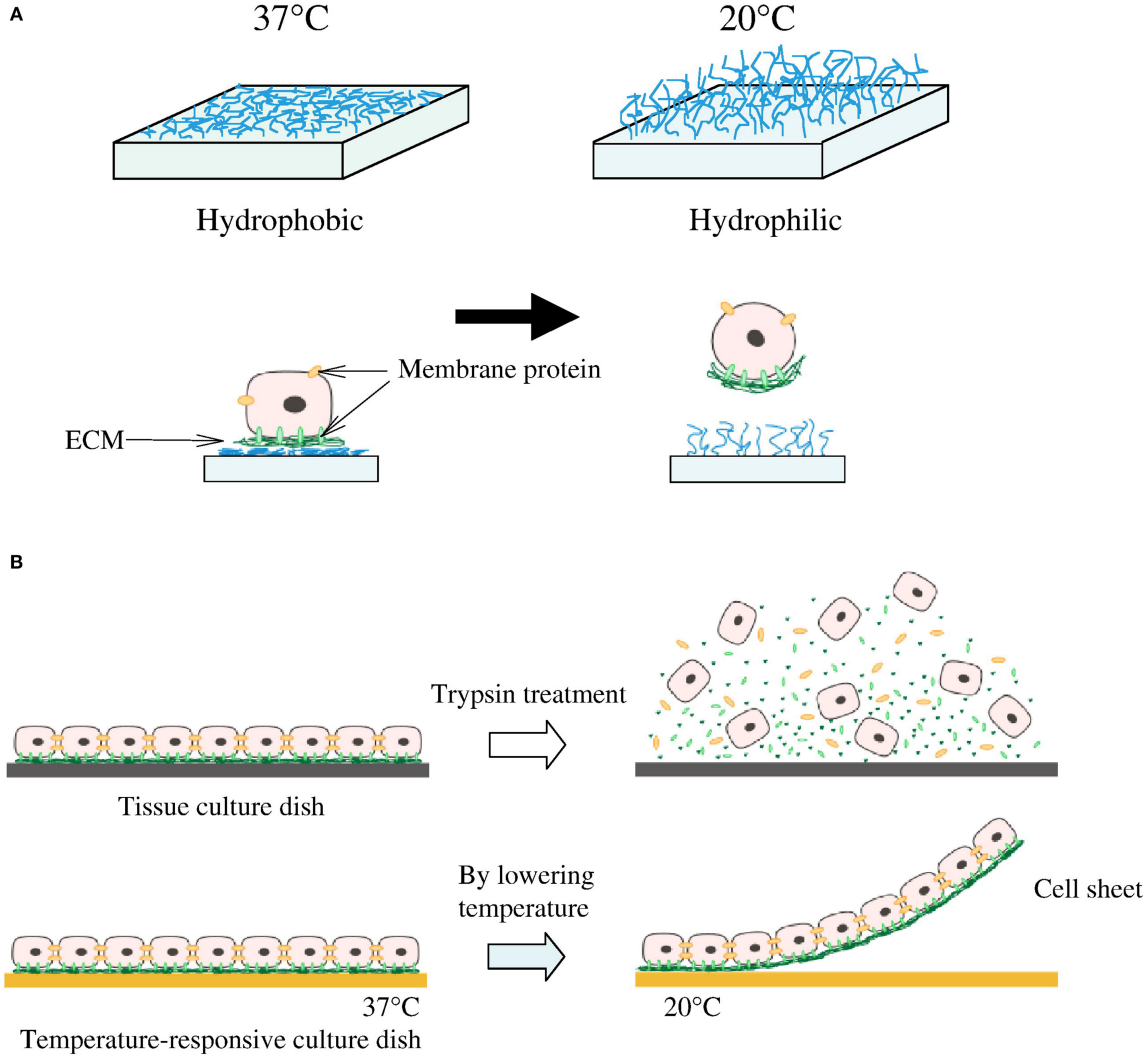
Cell sheeting. **(A)** At 37°C cells attach to the hydrophobic surface and at lower than 32°C detach from the hydrophilic surface. **(B)** Cells connect to each other by cell-to-cell junctions and ECM; when enzymes are introduced to remove cells from the surface, these junctions and ECM are disrupted. This is not the case for the temperature-responsive culture surface which is able to preserve the cell-to-cell junction and ECM. Reprinted from Moschouris et al. (87, 88).

The extension of 3D cultured scaffolds has also been applied toward cell-sheet technologies, where multiple layers of cells can be grown, transferred en bloc, and combined with varying cells to develop thicker and more complex tissue grafts, without scaffold use (Figure 2) (86, 88). Cells grown in biologically derived scaffolds can be exposed to a variety of growth factors that are intrinsic to the material, such as seen with Matrigel (89). However, even with synthetic scaffolds, various cytokines, miRNAs, and other cues can be spatially loaded to affect cell growth (90). What is most interesting is that scaffolds can be designed to permit differential cell growth, with regards to both variability and density. All these properties of 3D cultures/scaffolds make them of particular use in regenerative medicine and confer a significant benefit over 2D cultures in the development of complex tissue and structures.

But the scaffold is not the only environmental factor for bioengineered tissue; proper growth and maturation requires a unique combination of growth factors and differentiation signals provided by additional biomolecules (5). Over 300 ECM proteins, ECM-modifying enzymes and ECM-binding growth factors have been identified in mammalian cells as pivotal to growth, proliferation and regeneration processes (91). Of these, various collagens, proteoglycans and glycoproteins serve to provide strength, bind important growth factors, regulate protein complexes within tissues, promote cell adhesion, and participate in cellular signaling (83). There are further chemical signaling molecules, both autocrine and paracrine, utilized to promote proliferation and differentiation. The most critical for wound healing specifically are growth factors, namely fibroblast growth factors (FGF), epidermal growth factors (EGF), vascular endothelial growth factors (VEGF), transforming growth factor beta (TGF-beta), and platelet-derived growth factors (PDGF) (1, 92, 93). The combination of these proteins and growth factors initiates signaling cascades for hemostasis, inflammation, proliferation, angiogenesis, and wound healing (92, 93). Inclusion of these molecular and mechanical signals allow for engineered tissue cells to participate in multidirectional interactions within the tissue, as well as with the surrounding structures *in vivo* (94). Therefore, a proper microenvironment is crucial for clinically applicable bioengineering constructs. It is useful to note that many of these materials can be configured in a variety of ways utilizing a multitude of manufacturing approaches.

## Manufacturing

Within the field of regenerative engineering there are numerous manufacturing methods employed, most of which fall under three broad categories: decellularization, organoid technologies, and 3D bioprinting (Table 3). Each of the following methods have unique advantages for clinical application, as well as disadvantages for scale up capabilities.

**Table 3:** Summary of manufacturing methods.

**Method**	**Description**	**References**
Decellularization	• Utilizes washing, rinsing and sterilization of biological tissues to create a cell-free scaffold with embedded biomaterials • The decellularized extracellular matrix can then be re-cellularized *in vitro* or *in vivo* • Xenogeneic sourcing can address the issue of tissue scarcity • Approach has demonstrated some clinical translatability	(91–111)
Organoid technologies	• Small aggregates of cells secrete their own ECM to self-organize into 3D spheroids • Potential to recreate human organs in a nearly indistinguishable capacity • Utilized in drug testing and disease progression models • Not yet clinically relevant because of limited ability to recreate larger tissue models	(112–122)
3D Bioprinting	• Computer aided modeling and deposition of living cells, biomolecules, and materials into three-dimensional tissue • More precision than other manufacturing processes • Limited to avascular, aneural, thin, and hollow structures because of current printer capabilities • No widespread clinical translation to date	(3, 79, 123–126)

### Decellularization

**Figure 3:**
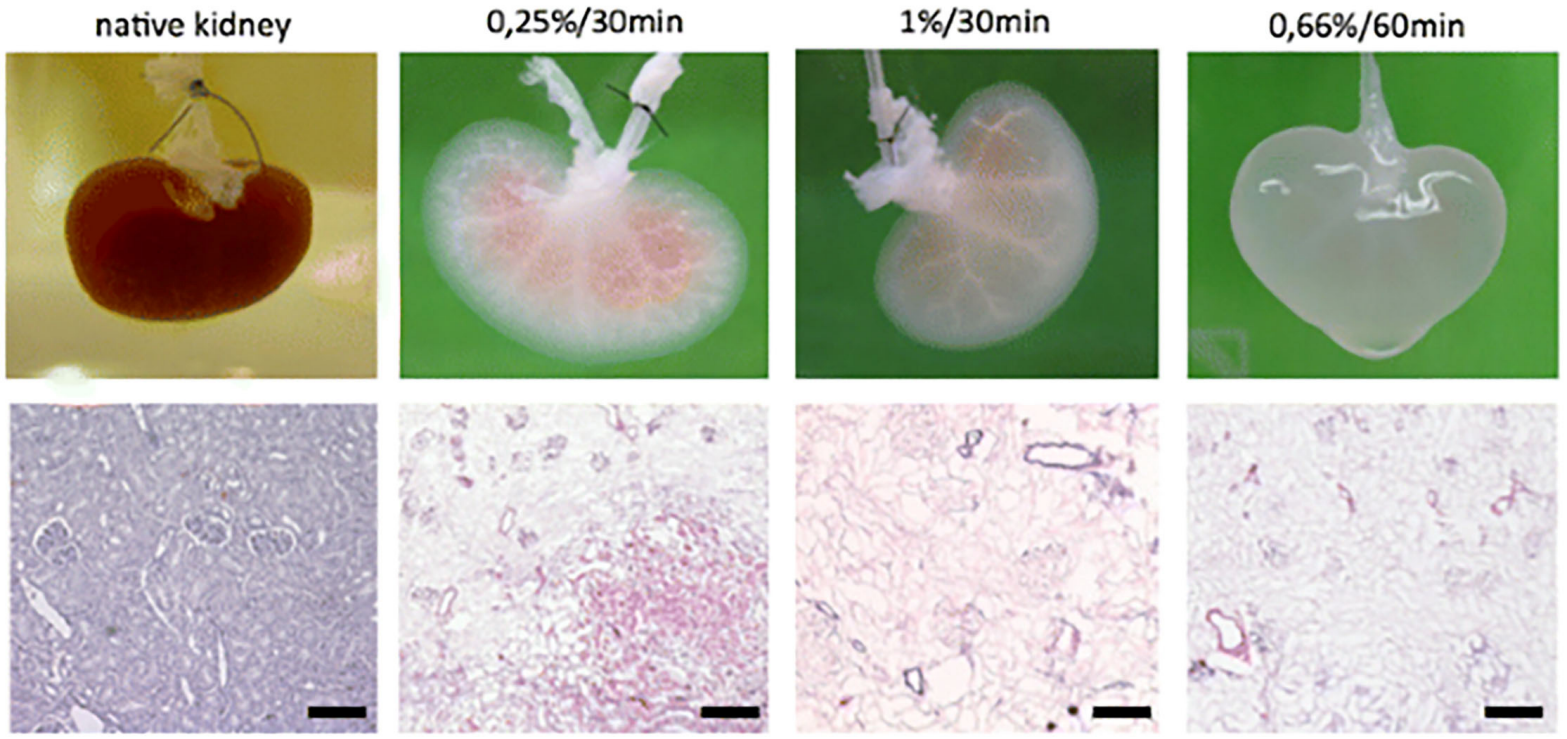
Macroscopic and histologic investigation of kidney decellularization efficacy of different SDS concentration/perfusion time combinations. Reprinted Schmitt et al. (100).

One method for ensuring not only a proper, but an ideal environment for tissue engineering, is through the decellularization of biological tissues. This process utilizes detergents to strip nuclear and cellular components from natural tissue, leaving behind the native architecture, growth factors and proteins, to produce a biological scaffold already embedded with biomaterials (95). As a versatile scaffold and biomaterial, decellularized matrices serve as a compatible platform for stem cell proliferation and specialization (96, 97). This approach can be utilized for “simple” decellularization resulting in the fabrication of thin structured matrices or whole organ decellularization in order to engineer more complex structures (98). The process of decellularization includes three phases: wash, rinse, and sterilization (Figure 3) (99, 100). During the wash phase, a detergent, enzyme, or denaturing agent is applied to the natural tissue in order to destroy cellular material and breakdown the tissue (101). The tissue is then rinsed, removing any residual detergent from the decellularized extracellular matrix (dECM) (102). Remaining detergent is potentially cytotoxic by inhibiting cell proliferation during the recellularization process (102). The last stage is sterilization, in which residual antigenic components of the donor tissue are removed from the dECM in order to prevent immunogenic responses by recipients of the final bioengineered product (103). The final dECM product can then be re-cellularized, either *in vitro* or *in vivo*. A variety of such products have also been used clinically, in applications such as breast reconstruction (104–109) and hernia repair (110–115) where *in situ* cellularization is relied upon. While the decellularization process overcomes certain challenges in tissue engineering, the need for a donor, fails to resolve one of the major hurdles in transplant surgery, tissue scarcity. However, xenogenic sourcing is an attractive way to remedy this, at least with modest tissue fragments.

### Organoid Technologies

**Figure 4:**
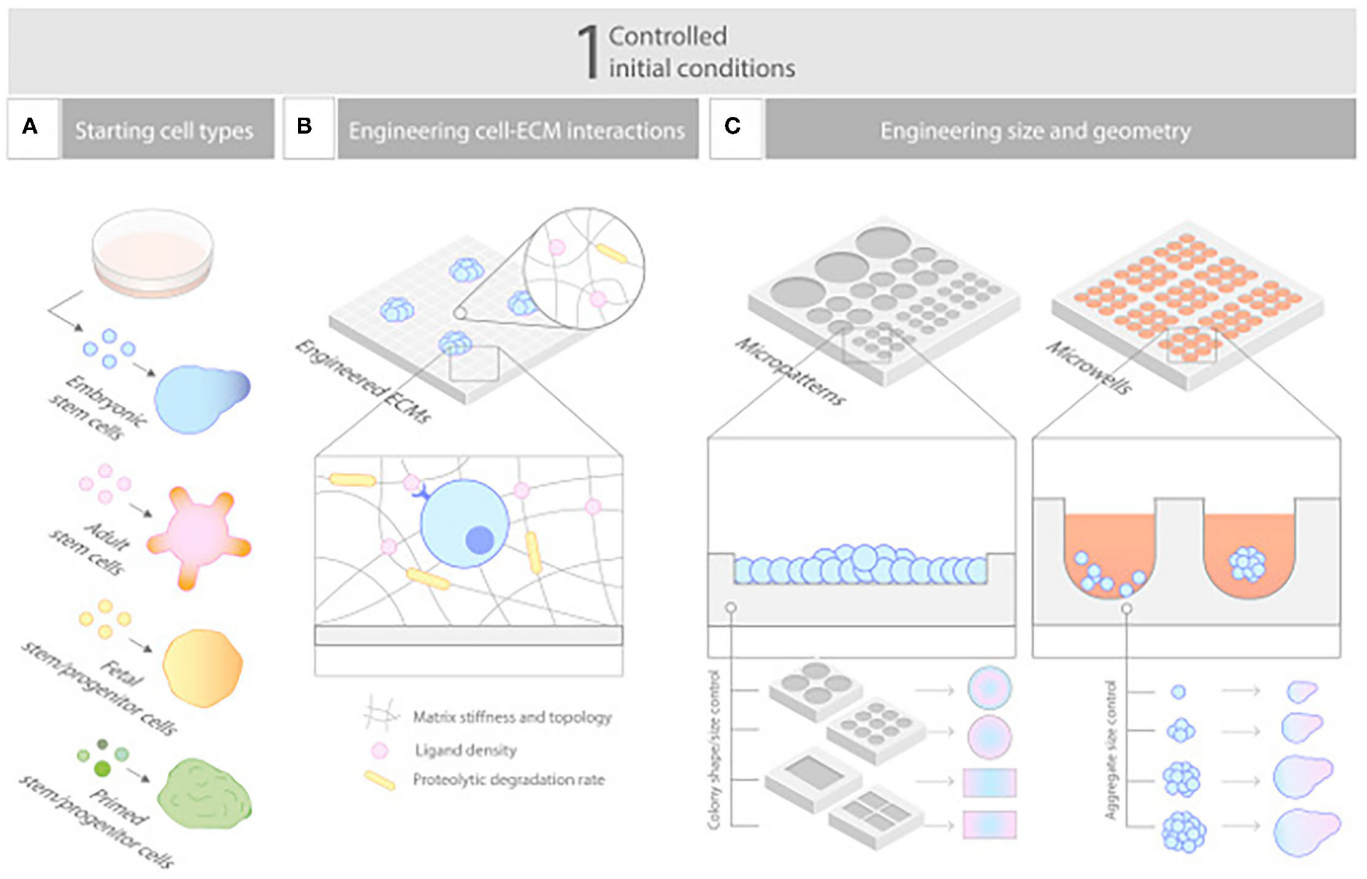
Controlling initial conditions. **(A–C)** Carefully designed starting conditions are critical for robust organoid formation, and these include **(A)** choosing the nature of the cells, **(B)** controlling their aggregation to a defined size and shape, and **(C)** engineering their environment to empower their ability to self-organize. Reprinted from Brassard and Lutolf (119).

Organoid technologies are 3D stem cell cultures able to reproduce human tissue both in structure and physiologic function, thereby, offering great potential for a personalized treatment strategy (116). As small aggregates of pluripotent or adult stem cells (117), organoids are able to self-organize into 3D spheroid structures, without the influence of foreign material (118). Like all *in vitro* models, these models confer a high degree of clinical and biological relevance (118), by mimicking tissue regeneration in a controlled environment (Figure 4) (119). However, what differentiates them from other *in vitro* models is their capability of recreating the histology and physiology of human organs in a nearly indistinguishable manner (120–126). This unique ability arises from stem cells secreting their own extracellular matrix and interacting with cells in their original microenvironment (118). The value of these small replicas of human tissue has increased quickly due to their function as drug testing and disease progression models, but they have numerous disadvantages when it comes to scale-up applications for bioengineering larger tissue and organs (118). As such, they have yet to find clinical relevance in surgical reconstructions (127).

### 3D Bioprinting

Bioprinting is the modeling of living cells, biomolecules, and biocompatible materials into three-dimensional functional tissue (83). These biologics are stacked and assembled in a computer-aided layer-by-layer seeding approach (128). 3D bioprinting is capable of a precision fabrication which was previously unavailable in traditional tissue engineering techniques. There exists the capability of exact positioning of various biologics and high digital control over speed, resolution, concentration, volume, and diameter of the cells being printed (3, 129). While this approach has numerous advantages, the method is limited by current bioprinter technology regarding cell concentration, drop volume, and diameter of printed cells; currently limiting its applicability to avascular, aneural, thin, and hollow tissues (3, 129). In recent years, several new methods have been developed to expand applicability of bioprinting, they can be classified into three major modalities based on their working mechanism: extrusion-based bioprinting (EBB), droplet-based, (DBB), and laser-based bioprinting (LBB) (130, 131). Each offers its own set of advantageous and disadvantages, signifying that widespread clinical translation is not yet on the immediate horizon. However, research is progressing rapidly across a multitude of diverse applications, such as breast reconstruction (132), craniomaxillofacial injuries (133), and cardiovascular repair (134).

## Preclinical Regenerative Engineering

In the past, 2D cell culture systems or animal models were used for both pharmaceutical screening and disease modeling, but due to their inability to properly mimic human tissues they have largely been replaced by bioengineered models (135–137). While 2D cell culture systems utilize the same cellular components as human tissue, they are unable to reproduce the complex 3D microenvironment of *in vivo* tissue (136, 138, 139). Conversely, animal models properly replicate the 3D structure of tissues and organs but their distinct multicellularity (140) decreases their efficacy in predicting human toxicology and adverse reactions (141). Therefore, bioengineered constructs have revolutionized medical research by providing a physiologic indistinguishable *in vitro* model of human tissue along with the ability to provide more accurate data on the pathophysiology of disease, drug efficacy, and drug toxicities (142). These simplified platforms provide us the capacity for preclinical testing of both specific disease entities as well as engineered constructs on a small scale.

### Drug Screening

Drug candidates' molecular interactions must be extensively studied with their associated biochemical target before market approval (3). Prior to the development of bioengineered tissue models, the use of *in vitro* toxicity and efficacy assays to accurately predict *in vivo* responses posed a major challenge to efficient and effective pharmacological advancement (3). However, organoid technologies have become a solution to this very challenge, by creating *in vitro* tissue and organ models that are structurally, cellularly, and physiologically indistinguishable from *in vivo* tissue (3). Skardal et al. proved this by testing a panel of medications that had passed studies using 2D *in vitro* cultures and animal models, only to be recalled once they were made available to the public secondary to significant toxicities and adverse reactions (143). These drugs were then retested using organoids, which were able to successfully demonstrate toxicity (143). Their study concluded that use of organoid models in drug testing could indicate both short and long-term toxicity. At present, organoids of all types of human tissues, including lung (144), heart (145), kidney, liver, and muscle tissue, have been utilized for this purpose (137).

### Disease Modeling

Similarly, organoids developed from either engineered cells or patient biopsy samples have been used to model disease for the study of infectious agents, genetic disorders, and malignancies (120). Lancaster et al. became the first researchers to utilize bioengineered tissue in this way in their study of microcephaly development using brain organoids in 2013 (123). A few years later, during the 2016 Zika epidemic, numerous studies related ZIKV to microencephaly using similar models (146–149). It has become apparent, that human organoid models are ideal for the study of infectious pathogenesis specifically, due to their tendency for species, or even tissue, tropism (120). Some examples of successful infectious disease research using organoid technology include: intestinal organoids for norovirus (150) and rotavirus (151, 152), airway organoids for respiratory viruses including RSV (153) and animal born influenza strains (154–156), co-cultivated human epithelia organoids in the study of bacteria and parasites including Helicobacter pylori (157), Cryptosporidium (158–160) and most recently in SARS-CoV-2 research to determine which organs the virus can infect and propagate in (161–163). The list of pathogens grown in organoid models is increasing rapidly (120).

Human organoid technologies are also becoming an increasingly important asset for precision medicine, by being able to directly test pathogenic genes and mutations in models derived directly from the patient (120). While this approach is still new, the most prominent example is the use of rectal organoids, developed from small endoscopic biopsies of Cystic Fibrosis patients to predict their response to more individualized medications and combined treatments (164–166).

Organoid models have also revolutionized the study of cancer, allowing researchers to study the mechanisms of angiogenesis, carcinogenesis, and metastasis in histologically and physiologically accurate *in vitro* models (167, 168). Using these models, researchers can appreciate the biochemical interactions leading to malignancies during replication, morphogenesis, differentiation, and growth, with the added ability of being able to control and change variables and environmental conditions (167). For example, numerous studies have validated the driver pathway mutations in tumorigenesis using gene-edited organoid models (169–172). Furthermore, it is believed that failure of most conventional cancer therapies is mainly due to cancer heterogeneity, meaning that the ability to reconstruct gene-mutation specific, and even patient-specific organoid models, is crucial for producing personalized cancer treatments (168). Thus far, cancer organoid models have been developed using patient samples from colon (173–177), brain (178, 179), prostate (180), pancreas (181–183), liver (184), breast (185), bladder (186), stomach (187–189), esophageal (190), endometrial (191), and lung tissues (153, 192).

## Clinical Applications

While large-scale clinical translation of bioengineered organs is years away, there have been several clinical applications that have improved patient care and become the standard of care, specifically in regenerative medicine (2). These successes are currently limited to thin and/or avascular tissues (130), including skin, cartilage, heart, and liver (193).

### Skin

The earliest documented clinical application of bioengineered tissue, in 1980, used fibroblasts, keratinocytes, and a scaffold to create skin tissue for wound healing (1). Since then, the use of engineered skin has been extensively studied, with researchers attempting to fabricate tissue capable of mimicking the natural healing process of skin, to accelerate wound healing as well as recovering function (194). These skin substitutes can serve as an alternative to conventional skin grafts when standard autologous replacement options are limited (195–198). In recent years, these bioengineered skin substitutes have become viable, mainstream wound healing option for patients, with numerous types and brands approved for clinical use by the Food and Drug Administration (FDA) (2). Furthermore, some bioengineered options have successfully promoted skin restoration in previously untreatable injuries (194).

**Figure 5:**
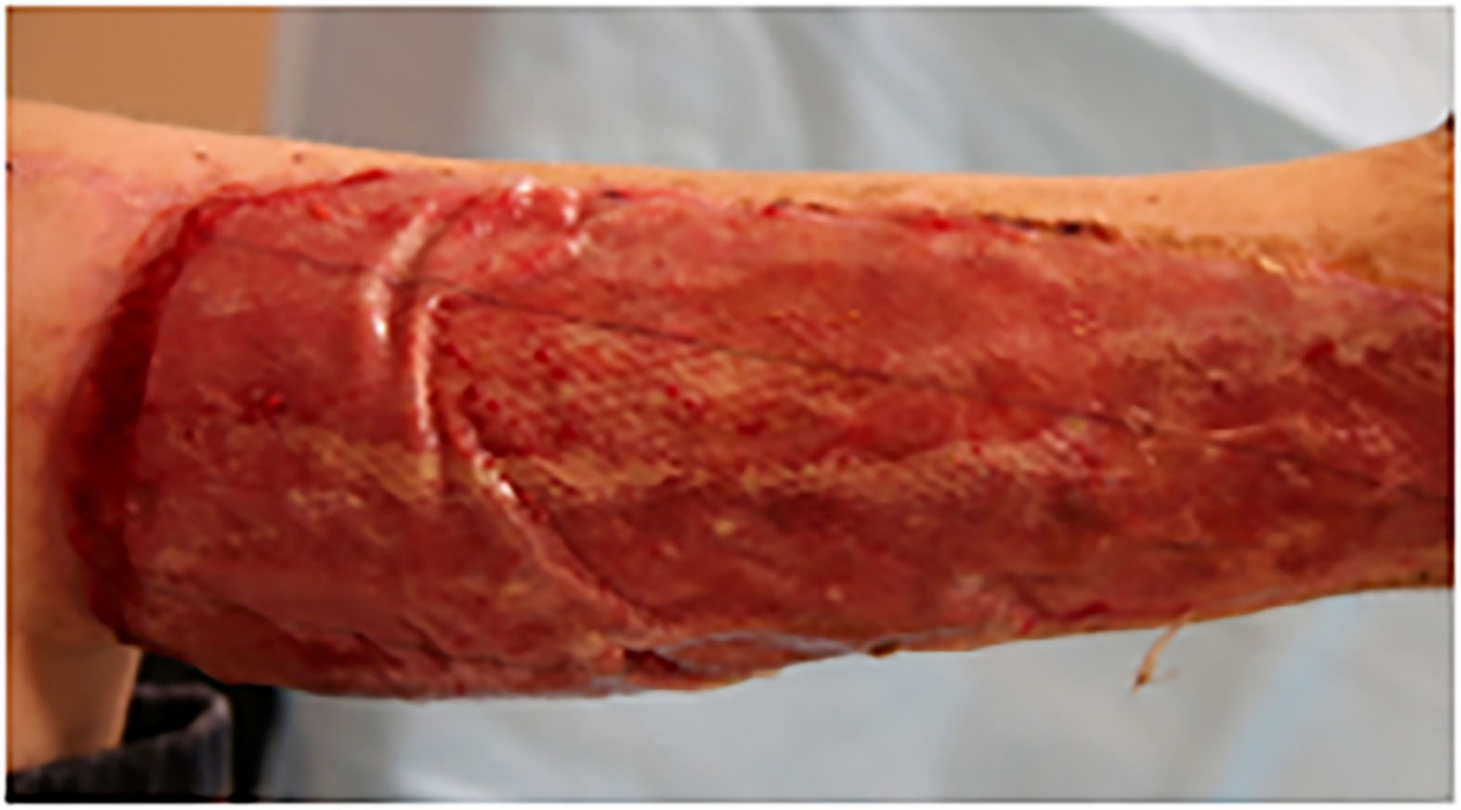
Collagen (type 1) dressing (Integra®, Plainsboro, NJ) for lower extremity wound repair.

The many bioengineered skin substitutes are classified based on both material origin (194), whether they are autologous, allogenic, or xenogeneic (198, 199); along with their cellularity (200). Different classifications have greater clinical utility depending on wound depth and whether a temporary or permanent dressing is needed (194). Acellular skin substitutes, like Integra®, utilize collagen and a silicon membrane in place of a dermis and epidermis, respectively (199) (Figure 5). As such, they are most applicable for superficial wounds or burns (199), providing a protective barrier against contamination and fluid loss, while also delivering dermal matrix components, cytokines and growth factors to promote natural wound healing (201, 202). Cellular skin substitutes are more complex, being composed of one to two layers of scaffold that are seeded with either autologous or allogeneic cells (200, 203). They enhance the healing process much like their acellular counterparts but are also capable of long-term and complete restoration of the injured tissue (204, 205). Allogeneic cellular skin substitutes, such as Dermagraft, are widely used, because they are not limited by donor availability (194). However, these are only suitable as a temporary wound dressing, and often are regrafted or replaced by either autologous cellular skin substitutes or split thickness skin grafts (194, 199, 206–210). Commercially available autologous options include Epicil®, Epidex®, and Tiscover® (A-Skin). These are more difficult to create, as grafts must include the full epidermal and superficial dermis layers to provide qualified cells (194). While these cultured epidermal autografts may be useful for long-term wound coverage (211, 212) they fail to provide the stability and resilience of native skin. Therefore, they are primarily used when there is a paucity of donor skin or when just the keratinocyte layer needs to be replaced (194).

### Cartilage

Unlike skin, cartilage lacks robust vasculature, nerves, and lymphatics. This often prevents natural healing *in vivo* (213). Furthermore, defects are difficult to repair due to insufficient chondrocyte supply (214). Until recently, repairing these common injuries required highly invasive, complicated, and imperfect solutions (215); leading the way for innovative bioengineered replacements (213). Within the field of bioengineered cartilage substitutes there are two main clinical applications being studied: joint and tracheal.

From the musculoskeletal perspective, successful bioengineered cartilage substitutes have currently only been developed with dimensions suitable for small joints (193). Clinically applicable examples include, Lee et al. generating vascularized, anatomically shaped tibial condyles (216). Scale-up for larger joints, like the hip and knee, has been limited due to vascular limitations and load bearing capabilities (193). Atala et al. developed a hybrid system using electrospinning and inkjet printing techniques to successfully produce scaffolds for cartilage tissue regeneration with greater mechanical and biologic properties (217). Still, while bioengineered cartilage repair was first introduced nearly three decades ago (218), current approaches are still unable to fabricate cartilage with zonal organization, ECM composition and mechanical properties that are indistinguishable from native tissue (219).

Bioengineered tracheal transplants were once considered the first large scale clinical application of tissue engineering; but ethical concerns and detrimental outcomes later caused much skepticism toward early successes (220). The three earliest reported cases of successful transplantation of tissue-engineered airways were published by the same research group, but with each utilizing a distinct approach (221). The first case produced a repopulated *ex vivo* decellularized tracheal allotransplant (222, 223). While initial analysis was promising, the transplant did not last long-term and the patient suffered numerous complications (224). In the second publication a synthetic tracheal prosthesis was repopulated using an *in vivo* tissue-engineering technique. However, this study was later retracted for unethical behavior in both clinical application and data reporting; after the patient in question developed serious complications and eventually died (225, 226). While the third case, utilizing a decellularized allotransplant with *in vivo* repopulation (227), was devoid of scandal, the researchers' association to the above cases caused skepticism within the medical community. Fortunately, in recent years, there have been several additional animal studies with encouraging results in tracheal bioengineering (228, 229).

### Heart

**Figure 6:**
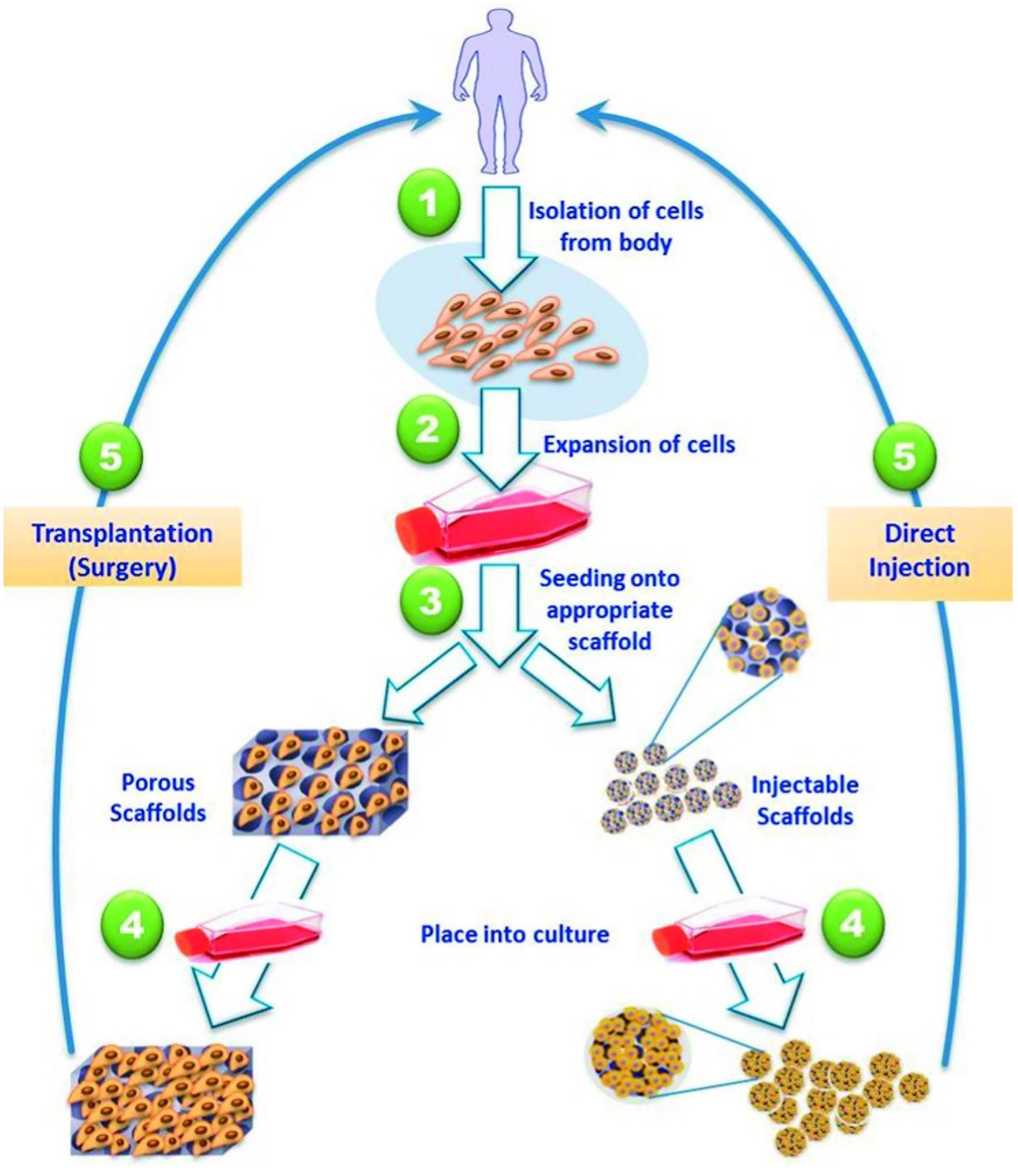
Strategies for tissue engineered cardiac replacement parts delivered either via surgical or percutaneous implantation. Reprinted from El-Sherbiny et al. (235).

Cardiovascular disease is a leading cause of worldwide morbidity and mortality affecting multiple anatomic sites. Although pharmacologic and surgical interventions have greatly improved therapeutic options; they are still incomplete. This can be appreciated across both congenital and acquired pathologies. For example, in disorders of the pediatric heart, such as septal defects, it would be optimal to have a replacement part which could grow with the child (230). However, in an adult who has suffered a myocardial infarction it is more beneficial to devise a thick patch that is contractile and able to propagate electrical currents (231). To fulfill these varied specifications, stem cells, and scaffolds have been used for cardiovascular repair over the past two decades and replacement parts have been generated. While the heart may house a limited amount of resident stem cells offering some innate regenerative capacity, other stem cell sources have proven more beneficial (232). This has led to mesenchymal (233), induced pluripotent, and hematopoietic stem cells being utilized for cardiovascular repair. For example, Matsumara described the usage of endothelial cells and bone marrow derived stem cells for scaffold seeding leading to the creation of tissue engineered vascular autografts (234). This approach has profound potential for the treatment of malfunctioning vessels, occluded arteries, and replacement hemodialysis access grafts. However, the *in vitro* generation of replacement parts, such as heart valves and blood vessels, requires cell isolation and expansion alongside scaffold fabrication followed by cell seeding and operative implantation (Figure 6) (235). This typically takes a substantial amount of time and effort, especially if autologous stem cells are utilized. Furthermore, the lack of an integrated vascular tree limits the potential of substantially thick tissues or whole organ generation (236). These limitations have led to the development of injectable therapeutics, where stem cells are introduced with or without the use of a carrier scaffold. A major advantage of an injectable is minimally invasive delivery, such as transcoronary infusions or transendocardial injections. This approach was first described in a rat model of myocardial infarction in which the survival of injectable myoblasts was significantly enhanced by a fibrin carrier (237). This may have been secondary to fibrin being a natural scaffold containing a variety of relevant growth factors. Since then, multiple innovative approaches have been described, such as alginate crosslinked with calcium delivered via a percutaneous catheter-based transcoronary infusion to reverse left ventricular remodeling following myocardial infarction in a swine model (238). Most of the scaffolds described have been hydrogel-based secondary to their structural similarities to native extracellular matrix and favorable environment for cell growth (235). Although hydrogel-based scaffolds may be better suited for injectable applications because of their malleability they can be modified and combined with other materials to provide the structural support required for *in vitro* tissue generation and surgical manipulation.

Due to the complex structure and function of the human heart, 3D bioprinting has proven promising in some current applications, due to its ability to effectively replicate native structural, mechanical, and functional properties of target tissues (83, 138, 239, 240). Currently, application of 3D bioprinted cardiac tissue is limited to myocardial patches and valves (3). Numerous distinct subsets of 3D bioprinting have successfully printed myocardial patches capable of beating spontaneously and undergoing action potential and uniform conduction, much like native ventricles (241). Laser Induced-Forward Transfer (LIFT) bioprinting was the first method to develop functional cardiac patches for clinical use (3). Testing proved enhanced angiogenesis in the border zone of infarction and greater retention of cardiac function post MI (242). Soon after, Gaetani et al. used extrusion printing to fabricate pre-vascularized myocardial patches that stimulated vascularization, and prolonged cell survival and tissue remodeling, after implantation (3, 243, 244). Further innovation by Tijore et al. increased contractile capabilities of their patches by fabricating microchanneled gelatin hydrogels (245).

While several traditional mechanical and bio-prosthetic valves have been used surgically for years, recent research has begun exploring 3D bioprinted valves (3). Current valve replacement options are limited by their durability, anticoagulation requirements and lack of patient specificity (3). Early studies hypothesized that bioengineered scaffolds have the potential to achieve increased elasticity (10-fold), improve shape retention and allow for greater cell viability (3). However, more research needs to be done regarding physiologic functionality in clinical applications (3). The further development of materials science and stem cell biology alongside enabling technologies will continue to improve tissue engineered replacement parts for cardiovascular repair, leading to improved patient outcomes.

### Bladder

Currently, the only complete human organ able to be bioengineered is the bladder, due to its thin, hollow structure. Yoo et al. produced the first successful canine example in 1998, by seeding a decellularized bladder submucosa with autologous cells (246). An equal number of subjects were implanted with either cell-seeded or acellular matrices, and it was discovered that the cell seeded group developed normal bladder tissue which was functionally and histologically indistinguishable from native tissue. Meanwhile, the acellular matrices developed into abnormal and functionally defective tissue (247). Within a year, Oberpenning et al. translated these results to humans using a biodegradable scaffold seeded with autologous cells (248). These were successfully implanted into patients, who continue to have moderately efficacious outcomes even 15 years later (247–249). This research is now in late stage human clinical trials using the same protocols and manufacturing steps as the original studies, with hopes of FDA approval in the near future (247).

## Hurdles

Despite tremendous advancement in regenerative engineering, there has been only limited clinical translation and important hurdles for future expansions remain, including both ethical and biological aspects. For example, *in vitro* tissue generation continues to be limited by requirements for long-term bioreactor maturation (250, 251), lacks the structural stability necessary for surgical manipulation, and is prone to significant hypoxia (252) until inosculation is achieved. Furthermore, as it is still arduous to fabricate an uninterrupted microvasculature that lengthens to an anastomosable vessel segment, all engineered cellularized constructs suffer some hypoxia upon implantation. This profoundly impedes our ability to generate thicker tissues (253). Hence, rapid vascularization continues to be a significant limiting factor in regenerative engineering.

### Integrating Vasculature Into Engineered Tissue

As isolated cells merged to form multicellular organisms, they were no longer able to obtain oxygen and remove waste via diffusion. Thus, the need for a circulatory system developed. Currently, tissue engineering is at the same crossroad. As mentioned above, current clinical application of bioengineered tissues and organs is limited to thin avascular tissues (2). The fundamental problem is the failure to manufacture microvascular networks able to both support tissue survival and withstand physiological pressures (4). While researchers have agreed for decades that a hierarchical vascular network is essential for successful scale-up of engineered tissues (254), it has persisted as a major limitation (255, 256).

### Importance of Vasculature in Engineered Tissue

Unlike larger vasculature, which can be identified as distinct anatomical entities, microvasculature, namely arterioles, capillaries, and venules, are structurally and functionally incorporated into the tissue they supply (128). These vessels are crucial for tissue survival, ensuring delivery of oxygen and essential nutrients, as well as the removal of metabolic waste (257, 258). Given that the tissue diffusion limit is ~100–200 μm (257, 259, 260), thicker tissues than this must have an integrated microvascular network to keep up with their high volumetric oxygen-consumption (255). Accordingly, thick organs like the liver, kidney, lungs, spleen, and heart are highly vascularized, and as such require formation of new vasculature once they grow past the diffusion limit (213, 261–263).

Despite the immense progress made in regenerative engineering, current technologies and methods are not yet able to produce large scale, thick, vascularized tissues, and organs (2, 213). With bioprinting, the main hurdle is the incapability to print sufficiently small structures. As the name implies, the microvasculature is miniscule in size, with luminal diameters of arterioles and venules averaging about 30 microns (10–200 microns) and capillaries averaging 5–10 microns (128). Current printer heads are unable of printing at this size, despite development of increasingly smaller nozzle sizes (131). While decellularization platforms can retain vascular structures, including micro-vessels, it faces the challenge of maintaining appropriate functionality (99). Thus far, the solutions used during the wash phase are too harsh for the treated vasculature to retain normal physiology (101).

### Current Approaches to Vascularize Engineered Tissues

Given that thick and complex organs are the most in demand for transplantation (>90%), namely liver, kidney and heart, overcoming the vascular limitation would revolutionize modern medicine (213). Currently, three different approaches have been largely studied to successfully vascularize engineered tissue for scaled-up applications: isolated 3D printing of the microvasculature, prevascularization, and the integration of oxygen generating particles.

There are several studies being done into the viability of separately bioprinting the microvasculature and tissue (255). Given that current 3D bioprinting technology is incapable of printing at the submicron scale, one proposed approach is to print vasculature at the smallest size possible, and allow for the natural progression of vascular anastomoses to create the desired capillary network (255). Two different methods have been explored: direct and indirect bioprinting. Direct bioprinting produces microscale neotissues through printing of organoids into an anatomical model and allowing the vasculature to self-assemble without the use of a scaffold (118, 255). Whereas, indirect printing uses a fugitive ink that is then removed by heat stimulated decrosslinking, leaving behind the vascular network (255).

Another proposed approach is the pre-vascularization of bioengineered tissue constructs, that allow an engineered tissue to rapidly integrate with host vasculature once implanted (254). The process of pre-vascularization requires encapsulating endothelial cells (ECs) or their progenitors, with other cell types *in vitro* to produce a ready-to-go microvasculature (254). In theory, once implanted this construct could quickly inosculate with the recipient and facilitate rapid perfusion (140, 258, 260). However, proper distribution of this vasculature, especially in thick tissue constructs, relies on *in vitro* cellular infiltration and self-organization (254), often resulting in slow and non-uniform vascular networks (264). Another limitation to this approach, is that for vascular self-assembly to occur, there must be a critical concentration of ECs seeded into the tissue construct, thereby limiting the co-culture number of other cells (264).

Given the capacity for angiogenesis, albeit slowly, another method to prevent initial hypoxia and cell death in implanted bioengineered tissues is the inclusion of oxygen generating particles. These particles can be designed to maintain a certain partial pressure of oxygen for a defined time period. With the goal of maintaining cell oxygenation and viability until vascularization has taken place from the recipient into the engineered graft. Oxygen generating particles can either be cerium oxide nanoparticles (CNPs), sometimes referred to as nanoceria, or silver nanoparticles (AgNPs) (265, 266). CNPs have been a successful therapeutic agent in tissue repair and regeneration because of its reactive oxygen species (ROS) properties, high angiogenic potential (265), and ability to induce stem cell differentiation (267). Similarly, AgNPs are also popular, due to their physiochemical properties generating ROS (266). However, they are less commonly used despite their therapeutic benefits, because of their potential toxicity to cells and tissues (266). However, it is well-appreciated that hypoxia is a profound angiogenic stimulator and it is possible that inclusion of these particles may actually delay vascularization of the implanted graft.

## Future Perspectives

Regenerative engineering is expanding briskly, with continued advances being made on the tangible platform that has developed over the past-quarter century. Most current approaches often rely heavily on *in vitro* manufacturing followed by implantation. However, newer technologies may allow for direct surgical repair. For example, intraoperative bioprinting would revolutionize surgical care (255). In theory, this technology would enable immediate regeneration of complex, large tissues in situ (255, 268, 269). Along with the technique itself, *in situ* bioprinting requires the development of simplified, portable bioprinters as well as scalable automated biofabrication production lines, which will need to be adequately explored prior to any attempt at clinical translation (130, 270, 271). *In situ* bioprinting, nevertheless, represents a promising future (130).

**Figure 7:**
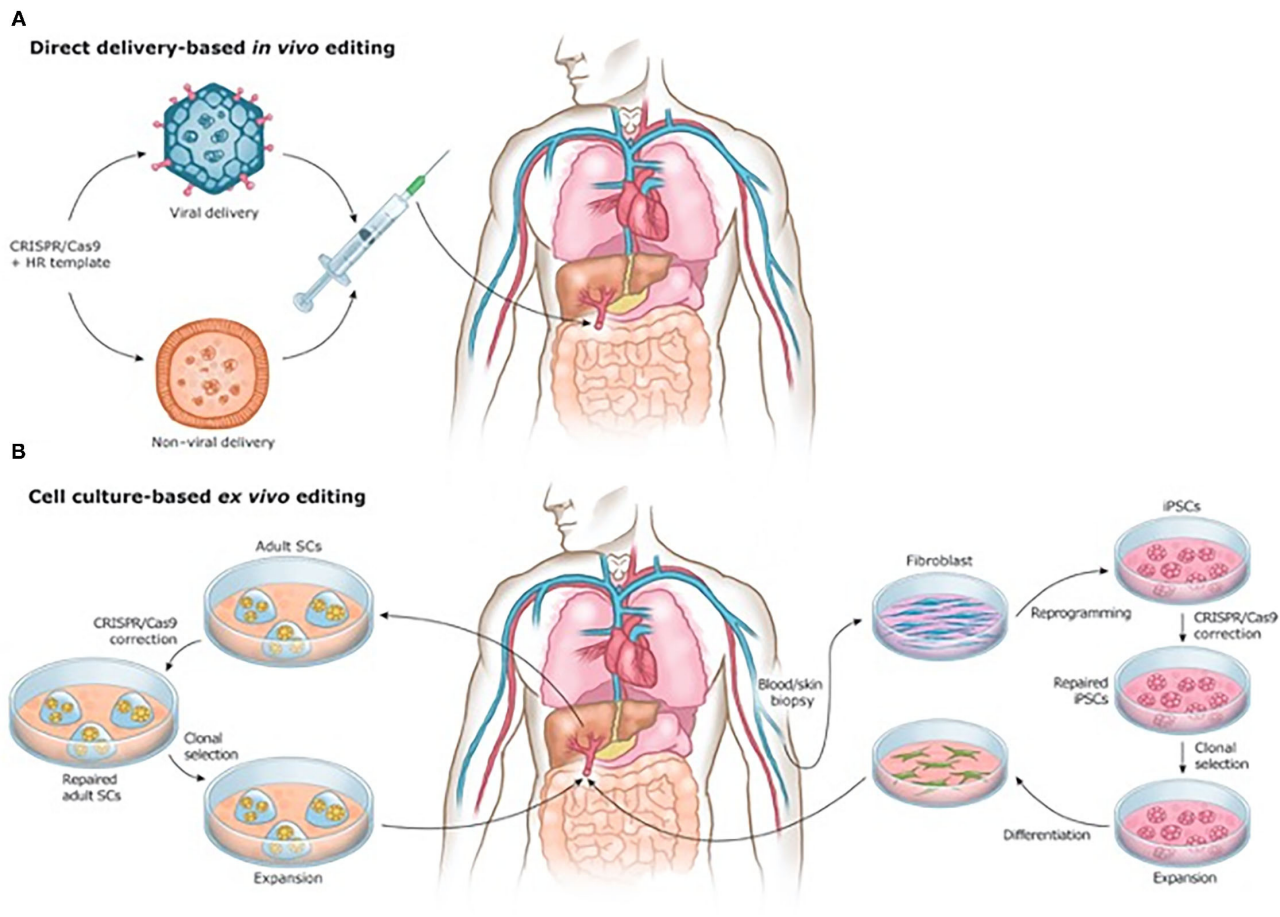
**(A)** *In vivo* and **(B)** *ex vivo* strategies for CRISPR/Cas9-based gene therapies. Reprinted from Savić et al. (276).

Beyond manufacturing, scale-up for tissue engineering is inhibited by the sheer multitude of cells comprising each organ, and the associated time constraints of developing organs on a patient-by-patient basis. It is estimated that each organ in the human body is made up of several billion cells (272). Meeting this need requires not only accelerating cell cycle speeds for increasing the quantity of stem cells produced and acquired, but also development of new materials or mechanisms capable of supporting differentiation of engineered tissue cells into specific phenotypes (131). Moreover, while current tissue engineering methods have proven successful in smaller, thin tissues and organs, the complex multicellularity of large, thick organs will require precise cell and biomaterial placement to ensure proper 3D structure, which has yet to be achieved on this large a scale (213). Additionally, further breakthroughs, such as cell encapsulation technologies (273), miRNA therapies and the clustered regularly interspaced short palindromic repeats (CRISPR) system of gene editing, may significantly alter the regenerative surgical landscape in years to come (Figure 7) (274–276).

## Conclusion

Regenerative engineering is a rapidly evolving field that combines the advances of tissue engineering with materials science, stem cell biology, and developmental biology. It has already produced clinically relevant technologies capable of improving patient outcomes and promises to continue revolutionizing the fields of medicine and surgery. Surgeons should become acquainted with some of its core components, as it will likely permeate into patient care, to a certain degree, within the foreseeable future.

## Author Contributions

All authors listed have made a substantial, direct and intellectual contribution to the work, and approved it for publication.

## Funding

This research was supported by the Pink Zone at the Pennsylvania State University (DJR), the American Association of Plastic Surgeons/Plastic Surgery Foundation Combined Grant (DJR) and the National Heart, Lung, and Blood Institute of the National Institutes of Health under Award Number R56HL 157190 (DJR).

## Conflict of Interest

The authors declare that the research was conducted in the absence of any commercial or financial relationships that could be construed as a potential conflict of interest.

## Publisher's Note

All claims expressed in this article are solely those of the authors and do not necessarily represent those of their affiliated organizations, or those of the publisher, the editors and the reviewers. Any product that may be evaluated in this article, or claim that may be made by its manufacturer, is not guaranteed or endorsed by the publisher.
